# The association between vitamin D status and tuberculosis in children: A meta-analysis

**DOI:** 10.1097/MD.0000000000012179

**Published:** 2018-08-21

**Authors:** Xiaoyun Gou, Lingli Pan, Fajuan Tang, Hu Gao, Dongqiong Xiao

**Affiliations:** a Emergency Department, West China Second University Hospital, Sichuan University; b Key Laboratory of Birth Defects and Related Diseases of Women and Children (Sichuan University), Ministry of Education, Chengdu, China.

**Keywords:** active tuberculosis, children, tuberculosis, vitamin D deficiency

## Abstract

**Background:**

Vitamin D deficiency (VDD) has been implicated in the pathogenesis of tuberculosis (TB), but most studies have not reported a significant association. We conducted a meta-analysis to explore the association between vitamin D status and TB in children.

**Methods:**

Web of Science, Ovid Medline, and EMBASE were searched for studies in English that discussed vitamin D status and TB in children before January 22, 2018.

**Results:**

From the 585 initially identified studies, we selected those that addressed an association between vitamin D status and TB according to our preselected inclusion criteria. Our meta-analysis included 10 studies. According to the random effects model, TB was significantly associated with VDD (ORs, 1.70; 95% CI, 1.20–2.42; *P* < .05) in children. Vitamin D levels were significantly lower in TB patients than in controls, with a mean difference *d* = −5.49 nmol/L (95% CI, −10.42 to −0.55; *P* < .05), indicating that VDD was significantly associated with TB (OR, 1.78; 95% CI, 1.30–2.44; *P* < .05) in children.

**Conclusion:**

This study suggests that vitamin D levels are significantly lower in children with TB/latent TB infection than in controls. TB may contribute to VDD in children. Therefore, VDD may be associated with TB in children.

## Abstract 2

Supplemental Digital Content is available in the text

## 1 Introduction

The definition of vitamin D deficiency (VDD) is inconsistent. Some studies define VDD as serum 25-hydroxyvitamin D3 (25(OH)D) levels less than 25 nmol/L (<10 ng/mL)^[1]^ and vitamin D insufficiency as 25(OH)D levels between 25 and 50 nmol/L (10–20 ng/mL); however, other studies define VDD as 25(OH)D levels less than 50 nmol/L (<20 ng/mL) and vitamin D insufficiency as 25(OH)D levels between 50 and 75 nmol/L (20–30 ng/mL).^[2,3]^ For humans, the sources of vitamin D are mainly sunlight exposure and vegetables.^[4]^ VDD is highly prevalent worldwide^[2]^ and leads to immune dysregulation and systemic inflammation.

The mechanisms underlying the association between VDD and tuberculosis (TB) may be as follows. Vitamin D is known to be essential for health and controls cell functions via vitamin D receptors (VDRs), which are present on myocytes, endothelial cells, and major immune cell types such as T and B lymphocytes, macrophages, dendritic cells, and neutrophils.^[1,5,6]^ Vitamin D regulates the immune system by modulating both innate and adaptive immunity.^[1]^ Various cytokines are expressed after the treatment of macrophages with 25(OH)D. Additionally, 2 antimicrobial peptides, cathelicidin and β-defensin, which stimulate autophagy in *Mycobacterium tuberculosis*, are expressed after exposure to vitamin D.^[1]^ Matrix metalloproteinases (MMPs) are inactive in the pathogenesis of pulmonary cavitation, and the process is induced by reactive nitrogen and oxygen via vitamin D. VDD may affect immunity, leading to an increased incidence of TB and sepsis.^[7]^ Williams et al found that VDD was common in children with active or latent TB, and sex, ethnicity, and TB status had no effect on vitamin D levels.^[4]^

TB, caused by *M tuberculosis*, is a leading health problem worldwide.^[2]^ One-third of the world's population is considered to have latent TB infection (LTBI).^[2]^ Several reports have shown an association between TB and malnutrition, demonstrating that multiple factors contribute to TB.^[8–10]^ Additionally, malnutrition affects the immune system and thus increases the risk of developing TB. In recent years, some studies^[11–13]^ have performed meta-analyses of the association between TB and VDD. Zeng et al^[11]^ showed that only 2/15 studies included in their meta-analysis included children. Most recent meta-analyses including patients of all ages did not present results for the subgroup of children.^[13,14]^ The studies presumed that the association between VDD and TB in children is limited. In addition, the sample sizes of the studies were small, and many of the studies lacked sufficient power to evaluate the relationship between vitamin D level, vitamin D status, and TB. Therefore, we conducted this meta-analysis to overcome these limitations.

## 2 Methods

### 2.1 Retrieval of studies

This meta-analysis followed the Meta-Analysis of Observational Studies in Epidemiology (MOOSE) and the Preferred Reporting Items for systematic Reviews and Meta-Analyses (PRISMA) guidelines. The Ovid Medline, EMBASE, and Web of Science databases were searched through January 22, 2018. The search consisted of the following 3 terms: child, TB, and vitamin D. We used the medical subject heading (MeSH) terms and key words to search for the first and second terms, and we used “AND” to connect child with TB. We used vitamin D and the relevant key words to search for the third term. In addition, we used “AND” to connect the third term and the combination of the first 2 terms. For MeSH terms and search strategy, see supplementary data. We restricted the search to human studies published in English. The retrieved studies were screened by reading the titles and abstracts, and 2 authors (XG and LP) independently read the full texts of the remaining publications and then discussed disagreements to reach a consensus.

### 2.2 Study selection

The study inclusion criteria were as follows: the participants were children <18 years old whose vitamin D levels had been measured so that the association between their vitamin D status and any of the following 3 outcomes could be investigated: the difference in the concentration of 25(OH)D (reported by the mean and standard deviation [SD]) between patients with active TB/LTBI and controls, the incidence, prevalence, relative risk (RR) or odds ratio (OR) of VDD in children with active TB/LTBI and controls, and the incidence, prevalence, RR, or ORs of TB/LTBI in children with VDD and controls; the study defined VDD; the study described the assessment of exposure and outcomes and reported unadjusted and/or adjusted RR and corresponding 95% confidence intervals (CIs) or unadjusted and/or adjusted ORs and 95% CIs; and the study was published in English, and the study design was case–control, cohort, or cross-sectional.

The exclusion criteria were as follows: the participants of the study were adults; the study was a meta-analysis, review, or case report; the study was not published in English; the article described an animal study; a study included data that overlapped with the data of another study; and the study did not have usable data (e.g., the concentration of 25(OH)D was not reported as the mean and SD but as the median and interquartile range [IQR], the controls were not truly free from TB infection, and the second or third outcomes with ORs or RR were not directly or indirectly available from the study).

### 2.3 Data extraction

The data were independently extracted from the studies by 2 reviewers (XG and LP) and included the following standardized forms: author, publication year, country, study population, study design, sample size, and outcomes.

### 2.4 Quality evaluation

The 2 reviewers (XG and LP) independently used the Newcastle-Ottawa Scale^[15]^ to examine all included studies for the methodological quality. The reviewers evaluated the quality score (with a maximum score of 9) in the following 3 domains: the study population selection, comparability, and evaluation of exposure and outcomes. The reviewers resolved disagreements as previously described.

### 2.5 Statistical analysis

The original included studies used the mean difference (MD) in 25(OH)D levels to evaluate the difference between the active TB/LTBI and control groups. We pooled the means and SDs of each study separately using the DerSimonian–Laird formula (random effects model).^[16]^ In addition, the original included studies used ORs and/or RRs and 95% CIs to assess the association between VDD and TB among the subjects. Both ORs and RRs were considered to be ORs. All data of the included studies were converted into log(ORs) and standard error (SE). We pooled the log(ORs) and SEs of each study separately using the DerSimonian–Laird formula (random effects model).^[16]^ Statistical heterogeneity^[17]^ between studies was assessed using the Q and *I*^2^ statistics. Values of *I*^2^ > 50% and *P* < .1 indicated a high degree of heterogeneity.^[18]^

We used funnel plots^[19]^ of SEs versus ORs to assess the publication bias and used Review Manager 5.3 software to perform the statistical tests.

## 3 Results

### 3.1 Literature search

**Figure 1:**
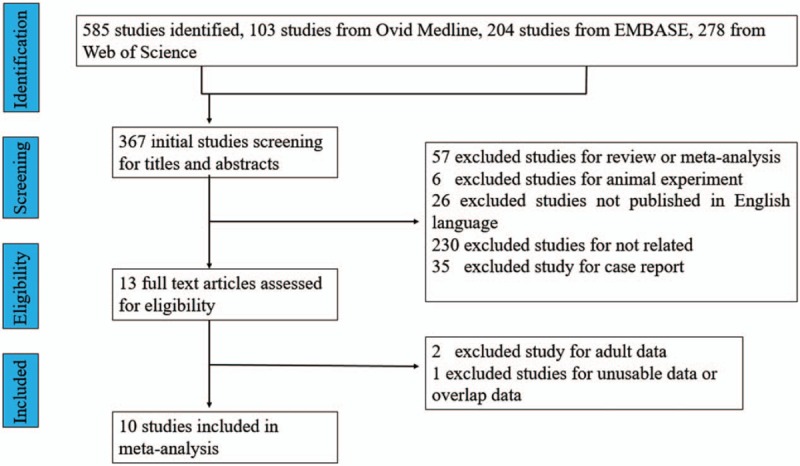
Flow chart for study selection.

We identified 585 potential studies including 103 from Ovid Medline, 204 from EMBASE, and 278 from Web of Science. After careful screening, 10 studies that reported the 25(OH)D levels or vitamin D status and presence of TB in children were included (see Fig. 1). These 10 included studies are summarized in Table 1.

**Table 1:**
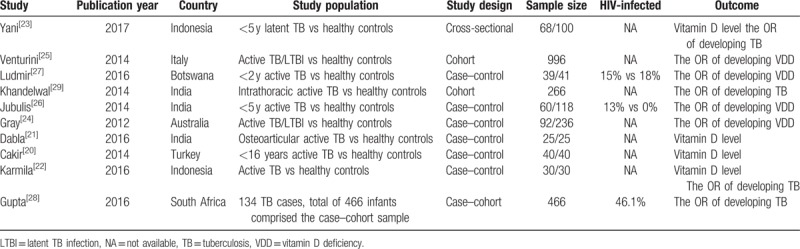
Characteristics of the included studies.

### 3.2 Characteristics and quality of the included studies

The included studies were published between 2012 and 2017. Four studies^[20–23]^ described the difference in 25(OH)D levels in children with TB and controls; two^[21,23]^ of these studies calculated 25(OH)D levels in nmol/L, and two^[20,22]^ calculated 25(OH)D levels in ng/mL. Four studies^[24–27]^ evaluated the association between the presence of any TB and VDD. One study^[25]^ showed a significant association between the presence of any TB and VDD, while 2 studies^[24,25]^ showed a significant association between the presence of active TB and VDD; however, 3 studies^[24,26,27]^ showed a nonsignificant association between the presence of any TB and VDD. Four studies^[22,23,28,29]^ showed that VDD contributes to TB.

## 4 Quantitative results (meta-analysis)

**Figure 2:**
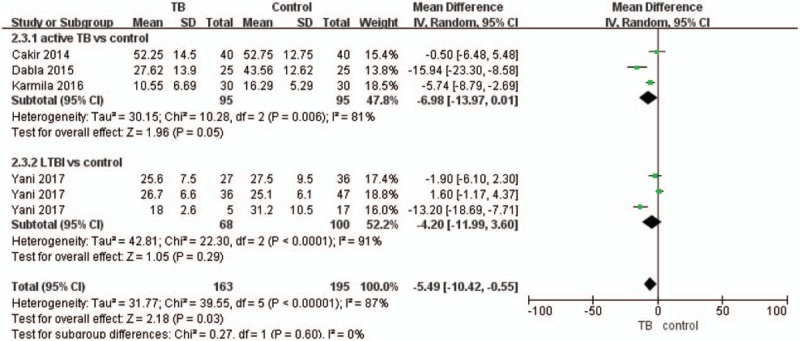
Forest plot of the comparison of vitamin D levels (nmol/L) between the active TB/LTBI and control groups. LTBI = latent TB infection, TB = tuberculosis.

**Figure 3:**
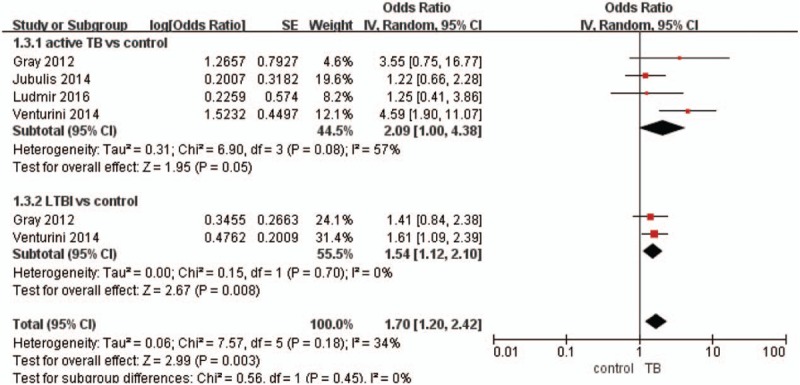
Forest plot of pooled odds ratios for vitamin D deficiency in the active TB/LTBI and control groups. LTBI = latent TB infection, TB = tuberculosis.

**Figure 4:**
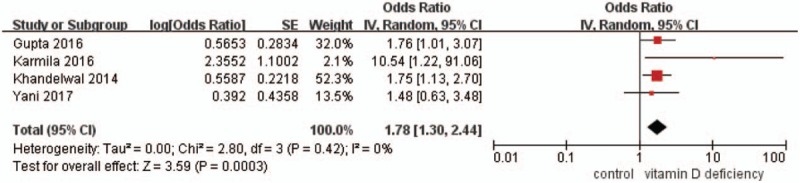
Forest plot of pooled odds ratios for tuberculosis in the vitamin D-deficient and vitamin D-sufficient groups.

The meta-analysis was performed using a random effects model (Figs. 2–4). Figure 2 shows that the MD in 25(OH)D levels (nmol/L) in children with TB and controls was −5.49 (95% CI, −10.42 to −0.55, *P* < .05, *I*^2^ = 87%), and the level of 25(OH)D was lower in the active TB/LTBI group than in the control group. The MD in 25(OH)D levels (nmol/L) between children with active TB or LTBI and controls was −6.98 (95% CI, −13.97 to 0.01, *P* = .05, *I*^2^ = 81%) and −4.20 (95% CI, −11.99 to 3.60, *P* = .29), respectively. The pooled data demonstrated that active TB was significantly associated with VDD, with a pooled OR of 2.09 (95% CI, 1–4.38, *P* = .05, *I*^2^ = 57%) (Fig. 3). Additionally, the pooled data demonstrated that LTBI was significantly associated with VDD, with a pooled OR of 1.54 (95% CI, 1.12–2.10, *P* < .05, *I*^2^ = 0%) (Fig. 3). Furthermore, the pooled data demonstrated that VDD was significantly associated with TB, with a pooled OR of 1.78 (95% CI, 1.30–2.44, *P* < .05, *I*^2^ = 0%) (Fig. 4).

### 4.1 Publication bias

Asymmetry and publication bias were evaluated by a funnel plot of the studies. The pooled results did not support the presence of significant publication bias (Supplementary Data).

## 5 Discussion

To the best of our knowledge, although several recent meta-analyses on TB have included children, none has reported the association between vitamin D status and TB/LTBI in children. This meta-analysis, which included 10 studies, provides evidence that vitamin D levels are significantly lower in children with TB/LTBI than in controls. TB/LTBI significantly contributed to VDD in children. Therefore, VDD was significantly associated with TB in children.

VDD might contribute to TB in children through multiple pathways. A growing body of evidence^[2,30]^ suggest that VDD affects immunity; it is possible that a low level of vitamin D may contribute to *M tuberculosis* infection by increasing chemokine production, activating dendritic cells, and altering T cell activation.^[11]^ Children with VDD in developing countries have a greater risk for developing TB.^[2]^ A recent meta-analysis^[13]^ showed that VDD in adults and children was associated with TB, with a pooled OR of 2.57 (95% CI, 1.74–3.80). However, the results of the studies focusing on the relationship between TB and VDD in children were inconsistent. Ludmir et al^[27]^ evaluated the relationship between active TB and VDD and identified an OR of 1.25 (95% CI, 0.41–3.86); however, Venturini et al^[25]^ demonstrated an association between TB and VDD, with an OR of 1.61 (95% CI, 1.09–2.39). Differences between studies may also be explained by interindividual differences due to VDR polymorphisms. The results of our meta-analysis showed that lower vitamin D levels were observed in children with TB than in controls. The definition of VDD varied from a 25(OH)D level <50 nmol/L (20 ng/mL) to <75 nmol/L (30 ng/mL) or <10 ng/mL, which may have contributed to the heterogeneity in the meta-analysis results. A consensus definition of VDD in children is thus required in future studies.

To better understand the relationship between TB and VDD in children, some studies have evaluated the association between active TB/LTBI and VDD. This meta-analysis demonstrated that the risk of developing VDD was prevalent in children with active TB and LTBI, with an OR of 1.70 (95% CI, 1.2–2.42). Furthermore, the results of this meta-analysis suggest that active TB increases the risk of VDD in children. Nevertheless, the analyzed studies did not include strict criteria for active TB, which may have led to an overdiagnosis of active TB and contributed to the heterogeneity among the studies.

Additionally, we found a significant association between VDD and active TB/LTBI. We found that VDD increased the risk of TB and that TB increased the risk of VDD. The lower level of 25(OH)D in children with active TB than in controls may be explained by poor nutrition or VDR polymorphisms. TB increases the risk of VDD via malnutrition, limited vitamin D absorption, limited sun exposure, immune dysregulation, and VDR polymorphism.^[1,28]^ Furthermore, the definition of VDD varied among studies, which may have contributed to the observed heterogeneity. Therefore, a consistent definition of VDD is required.

Other factors may interact with TB via pathogenetic pathways leading to VDD in children. Although the included studies controlled for some confounding variables, many other factors that contribute to VDD in children could not be excluded. For example, HIV-infected subjects and malnourished subjects are more susceptible to active TB/LTBI^[26,28]^ due to immune dysregulation. Jubulis et al^[26]^ and Gupta et al^[28]^ found that HIV status was a significant predictor of TB.

Similarly, some factors may interact with VDD via pathogenetic pathways to cause TB in children. Although the included studies controlled for some confounding variables, many other factors that contribute to TB in children could not be excluded.

Our meta-analysis has some limitations. First, we included only articles published in English, which may have contributed to publication bias. Second, the results of this meta-analysis should be interpreted with caution because of the limited number of subjects. Third, the bias inherent in observational studies was not eliminated in a quantitative synthesis. Additionally, the differences in design among studies may have affected the pooled results of the studies. Finally, the 25(OH)D level can be affected by many factors, and the measurement of 25(OH)D may not directly reflect vitamin D status.

Our meta-analysis also has several merits. First, we evaluated the association between vitamin D status and TB in children. Second, we demonstrated that the lack of a uniform definition of VDD contributes to heterogeneity among studies and that a consistent definition of VDD is required for future studies.

The results of our study indicate that supplementation with vitamin D may be beneficial for active TB/LTBI treatment and prevention, although the few relevant trials that have been conducted have had disappointing results.^[31–34]^

## 6 Conclusions

In conclusion, our pooled analyses provide strong evidence that vitamin D levels are significantly lower in children with TB/LTBI than in controls. TB may contribute to VDD in children, and thus, VDD may be associated with TB in children. This knowledge may support the design and evaluation of randomized case–control clinical trials on the role of vitamin D supplementation in preventing TB in children.

## Author contributions

XG and LP contributed to the conception and design of the study, as well as to the drafting of this article. FT and HG contributed to the collection and analysis of the data. DX contributed to the conception and design of the study and approved the final version of the manuscript for publication. In addition, we would like to thank American Journal Experts (www.aje.com) for editing the English language of the manuscript.

**Conceptualization:** Xiaoyun Gou, Lingli Pan, Dongqiong Xiao.

**Data curation:** Xiaoyun Gou, Lingli Pan, Dongqiong Xiao.

**Formal analysis:** Xiaoyun Gou, Lingli Pan, Fajuan Tang, Hu Gao, Dongqiong Xiao.

**Investigation:** Xiaoyun Gou, Fajuan Tang, Hu Gao.

**Methodology:** Xiaoyun Gou, Hu Gao, Dongqiong Xiao.

**Project administration:** Xiaoyun Gou, Lingli Pan, Dongqiong Xiao.

**Resources:** Xiaoyun Gou, Fajuan Tang.

**Software:** Xiaoyun Gou, Lingli Pan, Fajuan Tang, Hu Gao.

**Supervision:** Xiaoyun Gou, Lingli Pan, Fajuan Tang, Hu Gao, Dongqiong Xiao.

**Visualization:** Xiaoyun Gou, Fajuan Tang.

**Writing – original draft:** Xiaoyun Gou, Lingli Pan.

**Writing – review & editing:** Dongqiong Xiao.

## Supplementary Material

Supplemental Digital Content
