# Supplementary material for: The association between vitamin D status and tuberculosis in children: A meta-analysis
Source: Medicine (Baltimore). 2018 Aug 21;97(35):e12179. doi: 10.1097/MD.0000000000012179 (PMC6392646; doi:10.1097/MD.0000000000012179)

**The association between vitamin D status and tuberculosis in children: A meta-analysis**

Xiaoyun Gou^1,2#^, Lingli Pan^1,2#^, Fajuan Tang^1,2^, Hu Gao^1,2^, Dongqiong Xiao^1,2^*

**Supplement 1.**

Before 22 January 2018.

**Search strategy of Ovid 103**

1. exp Vitamin D/

2. vitamin D.tw.

3. vitamin D2.tw.

4. vitamin D3.tw.

5. 1-alpha hydroxyvitamin D3.tw.

6. 1-alpha-hydroxy-vitamin D3.tw.

7. 1-alpha hydroxycalciferol.tw.

8. 1-alpha-hydroxy-calciferol.tw.

9. 1,25 dihydroxyvitamin D3.tw.

10. 1,25-dihydroxy-vitamin D3.tw.

11. 1,25 dihydroxycholecalciferol.tw.

12. 1,25-dihydroxycholecalciferol.tw.

13. 25-hydroxycholecalciferol.tw.

14. 25 hydroxycholecalciferol.tw.

15. 25 hydroxyvitamin D.tw.

16. 25-hydroxy-vitamin D.tw.

17. alfacalcidol.tw.

18. calcidiol.tw.

19. calcitriol.tw.

20. calcifediol.tw.

21. calciferol.tw.

22. ergocalciferol.tw.

23. cholecalciferol.tw.

24. or/1-23

25. (child$ or infant$ or babies or baby or toddler$ or girl$ or boy$ or pre-school$ or preschool$ or nurser$ or kindergarten$ or kinder-garten$).tw.

26. (tuberculosis or tuberculos*).tw.

27. 24 AND 25 AND 26

**Search strategy of EMBASE:204**

1. exp Vitamin D/

2. vitamin D.tw.

3. vitamin D2.tw.

4. vitamin D3.tw.

5. 1-alpha hydroxyvitamin D3.tw.

6. 1-alpha-hydroxy-vitamin D3.tw.

7. 1-alpha hydroxycalciferol.tw.

8. 1-alpha-hydroxy-calciferol.tw.

9. 1,25 dihydroxyvitamin D3.tw.

10. 1,25-dihydroxy-vitamin D3.tw.

11. 1,25 dihydroxycholecalciferol.tw.

12. 1,25-dihydroxycholecalciferol.tw.

13. 25-hydroxycholecalciferol.tw.

14. 25 hydroxycholecalciferol.tw.

15. 25 hydroxyvitamin D.tw.

16. 25-hydroxy-vitamin D.tw.

17. alfacalcidol.tw.

18. calcidiol.tw.

19. calcitriol.tw.

20. calcifediol.tw.

21. calciferol.tw.

22. ergocalciferol.tw.

23. cholecalciferol.tw.

24. or/1-23

25. (child$ or infant$ or babies or baby or toddler$ or girl$ or boy$ or pre-school$ or preschool$ or nurser$ or kindergarten$ or kinder-garten$).tw.

26. (tuberculosis or tuberculos*).tw.

27. 24 AND 25 AND 26

**Search strategy of Web of science:278**

TS=(vitamin D or vitamin D2 or vitamin D3 or 1-alpha hydroxyvitamin D3 or 1-alpha-hydroxy-vitamin D3 or 1-alpha hydroxycalciferol or 1-alpha-hydroxy-calciferol or 1,25 dihydroxyvitamin D3 or 1,25-dihydroxy-vitamin D3 or 1,25 dihydroxycholecalciferol or 1,25-dihydroxycholecalciferol or 25-hydroxycholecalciferol or 25 hydroxycholecalciferol or 25 hydroxyvitamin D or 25-hydroxy-vitamin D or alfacalcidol or calcidiol or calcitriol or calcifediol or calciferol or ergocalciferol or cholecalciferol)

TS=(child or infant or child$ or infant$ or babies or baby or toddler$ or girl$ or boy$ or pre-school$ or preschool$ or nurser$ or kindergarten$ or kinder-garten$)

TS=(tuberculosis or tuberculos*)

AND the TS above

**Supplement 2. Newcastle - Ottawa Quality Assessment Scale results for case-control studies**

| Question | **Option** | Yani | Ludmir | Jubulis | Gray | Dabla | Cakir | Karmila |
| --- | --- | --- | --- | --- | --- | --- | --- | --- |
| Is the case definition adequate? | a) yes, with independent validation *****  b) yes, eg record linkage or based on self reports  c) no description | a | a | a | a | a | a | a |
| Representativeness of the cases | a) consecutive or obviously representative series of cases *****  b) potential for selection biases or not stated | b | b | a | b | a | a | b |
| Selection of Controls | a) community controls *****  b) hospital controls  c) no description | b | b | b | b | b | b | b |
| Definition of Controls | a) no history of disease (endpoint)*****  b) no description of source | a | a | a | a | a | a | a |
| Comparability of cases and controls on the basis of the design or analysis | 1. study controls for (Select the most important factor.***** 2. study controls for any additional factor (This   criteria could be modified to indicate specific  control for a second important factor.) ***** | a | a | a | a | a | a | a |
| Ascertainment of exposure | a) secure record (eg surgical records)*****  b) structured interview where blind to case/control status*****  c) interview not blinded to case/control status  d) written self report or medical record only  e) no description | a | a | a | a | a | a | a |
| Same method of ascertainment for cases and controls | a) yes*****  b) no | a | a | a | a | a | a | a |
| Non-Response rate | a) same rate for both groups*****  b) non-respondents described  c) rate different and no designation | b | b | b | b | b | b | b |

**Supplement 3. Newcastle - Ottawa Quality Assessment Scale results for cohort studies**

| Question | **Option** | Venturini | Khandelwal | Gupta |
| --- | --- | --- | --- | --- |
| Representativeness  of the exposed cohort | 1. truly representative of the average   (describe) in the community *   1. somewhat representative of the   average in the community*   1. selected group of users eg nurses,   volunteered) no description of the  derivation of the cohort | b | b | b |
| Selection of the  Non-exposed cohort | 1. drawn from the same community as   the exposed cohort*   1. drawn from a different source 2. no description of the derivation   of the non-exposed cohort | a | a | a |
| Ascertainment of exposure | a) secure record (eg surgical records)*  b) structured interview*  c) written self report  d) no description | a | a | a |
| Demonstration that outcome  of interest was not present at start of study | a) yes*  b) no | a | a | a |
| Comparability of cohorts on the basis of the design or analysis | 1. study controls for _   (select the most important factor)*  b) study controls for any additional factor* | a | a | a |
| Assessment of outcome | a) independent blind assessment*  b) record linkage*  c) self report  d) no description | b | b | b |
| Was follow-up long enough  for outcomes to occur | a) yes*  b) no | b | a | a |
| Adequacy of follow up  of cohort | 1. complete follow up - all subjects   accounted for*   1. subjects lost to follow up unlikely   to introduce bias - small number  lost - > _ % (select an adequate %) follow up, or description provided of those lost)  c) follow up rate < _% (select an adequate %) and no description of those lost  d) no statement | d | d | d |

**Supplement 4.** Funnel plot of comparison of vitamin D level between tuberculosis and control group.


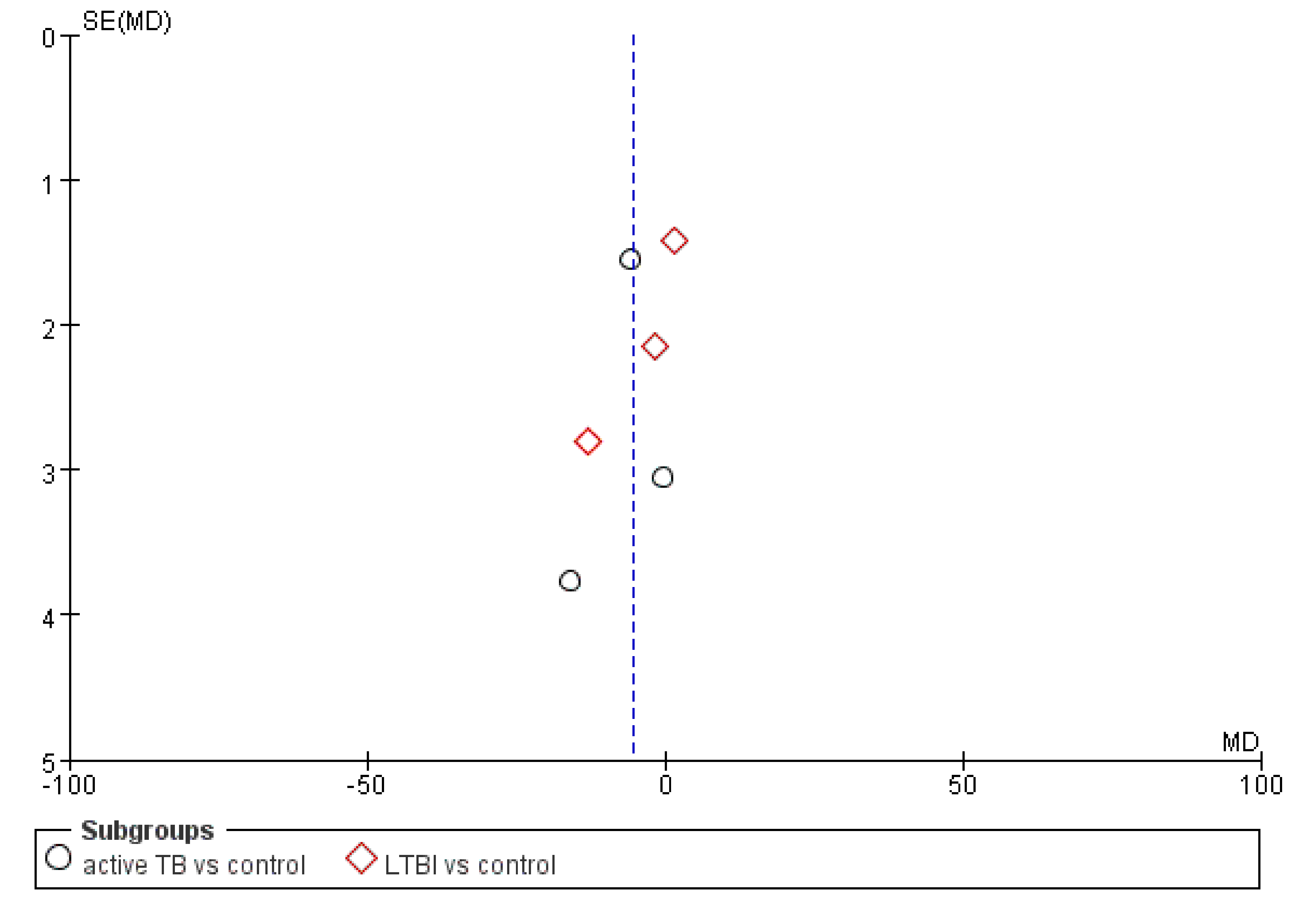


**Supplement 5.** Funnel plot of pooled odds ratio of vitamin D deficiency in children with TB and control.


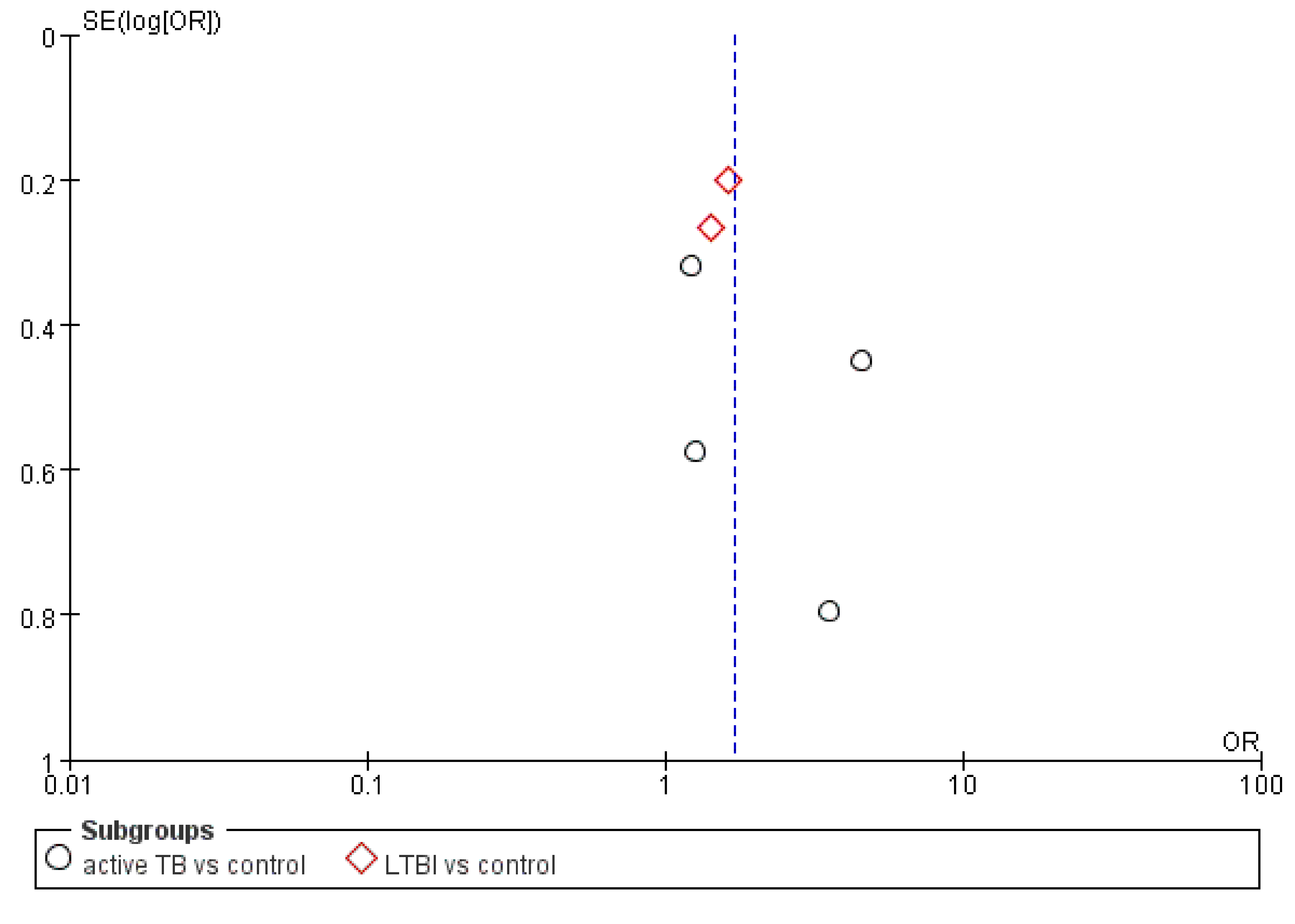


**Supplement 6.** Funnel plot of pooled odds ratio of TB in children comparing vitamin D deficiency and sufficiency.


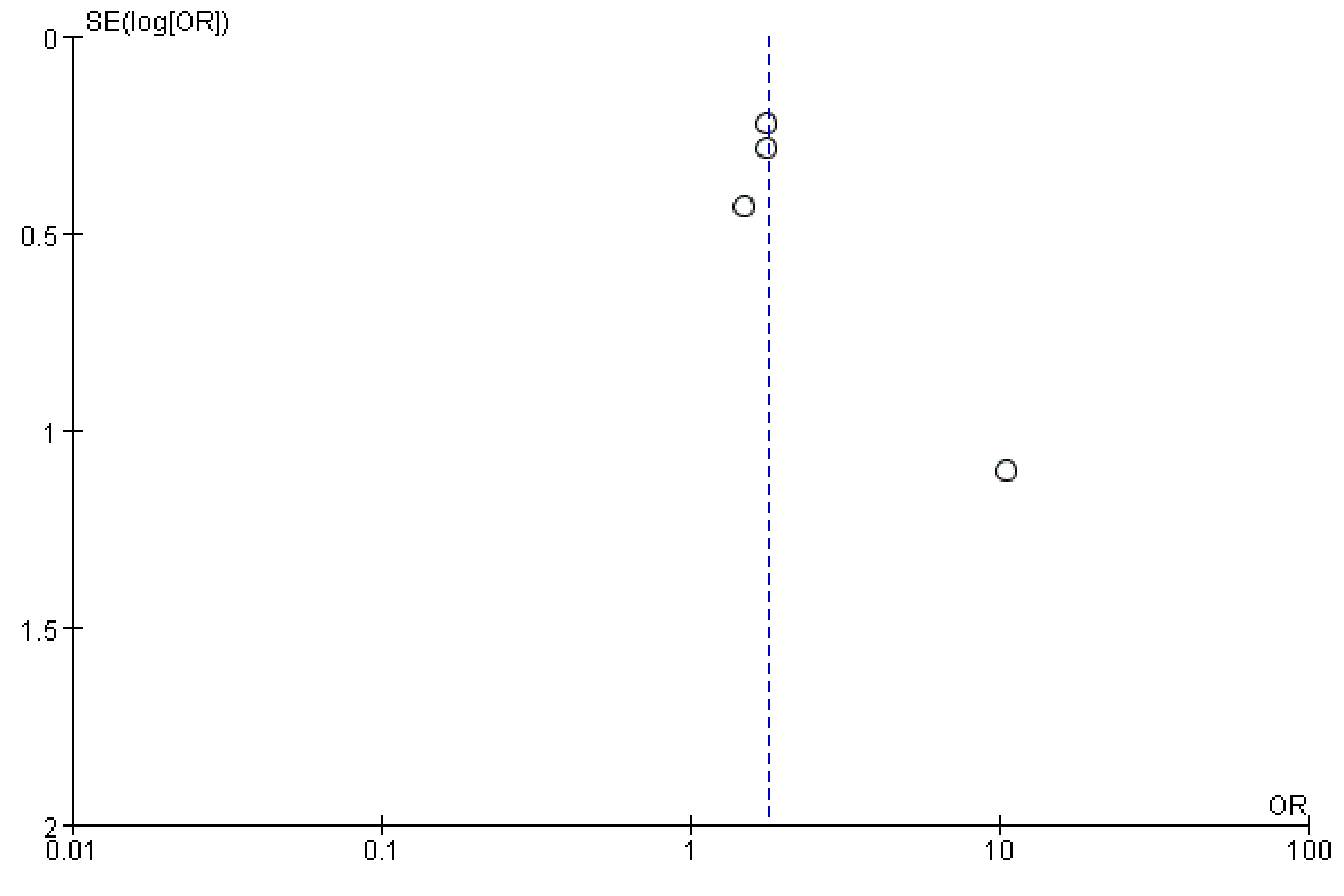

Supplement: Supplemental Digital Content [file medi-97-e12179-s001.docx]
